# The Effect of Listening to Holy Quran Recital on Pain and Length of Stay Post-CABG: A Randomized Control Trial

**DOI:** 10.1155/2023/9430510

**Published:** 2023-11-06

**Authors:** Mohannad Eid Aburuz, Ghadeer Al-Dweik, Fatma Refaat Ahmed

**Affiliations:** ^1^Clinical Nursing Department, Faculty of Nursing, Applied Science Private University, Amman, Jordan; ^2^Nursing Administration, Faculty of Nursing, Applied Science Private University, Amman, Jordan; ^3^Department of Nursing, College of Health Sciences, University of Sharjah, Sharjah, UAE; ^4^Critical Care and Emergency Nursing, Alexandria University, Alexandria, Egypt

## Abstract

**Background:**

Nearly, 75% of patients post-CABG complain of moderate to severe pain during their hospital stay. Nonpharmacological interventions have been investigated; however, the effect of Holy Quran recital post-CABG is still not well studied, especially in developing Islamic countries.

**Objective:**

To investigate the effect of listening to the Holy Quran recital on pain and length of stay post-CABG.

**Methods:**

This was a randomized control trial on 132 patients recruited from four hospitals in Amman, Jordan. The intervention group listened to the Holy Quran recited for 10 minutes twice daily while the control group received the usual care. Data were analyzed using paired and independent samples *t*-tests.

**Results:**

Paired *t*-test testing showed that there was a significant reduction in the pain level, (M [SD], 6.82 [2.27] vs. 4.65 [2.18], *t* = 23.65, *p* < 0.001) for the intervention group. In addition, the intervention group had shorter LoS in the ICU and in the hospital compared to the control group, (M [SD], 5.0 [4.02] vs. 6.58 [4.18], *t* = −2.1, *p* < 0.05), (M [SD], 10.15 [9.21] vs. 15.01 [13.14], *t* = −2.6, *p* < 0.05), respectively.

**Conclusions:**

Listening to the Quran was significantly effective in improving pain intensity among post-CABG patients and shortening their hospital/ICU stay. This trial is registered with NCT05419554.

## 1. Introduction

Open-heart surgery is currently carried out in industrialized and developing nations to extend patient lives and enhance the quality of life [1, 2]. The most frequent open-heart procedure is a coronary artery bypass graft (CABG) [2, 3] which accounts for around 60% of all cardiac operations [2]. Heart surgery, despite being a successful cardiac treatment, is nevertheless a traumatic procedure that can result in a variety of side effects, such as excruciating pain at the surgical site, stroke, pulmonary edema, pericarditis, and postoperative depression [2, 4, 5]. For this reason, patients are transferred to the intensive care unit (ICU) following CABG surgery and are monitored using a variety of medical devices, including electrocardiogram monitors, arterial and central lines, ventilators, and chest tubes.

It has been reported that up to 75% of patients complain of moderate to severe pain in the first 48 hours post-CABG [2, 6]. The sternotomy, saphenous vein, and internal thoracic artery harvesting, as well as the insertion of numerous drains, are some of the variables that lead to pain after CABG surgery [7]. Pain stimulates the sympathetic nervous system which in turn increases the secretion of both epinephrine and norepinephrine causing hypertension, tachycardia, and tachypnea. Furthermore, pain increases the workload of the heart making an imbalance between oxygen supply and demand. Consequently, ventricular dysfunction and ischemia might occur [8, 9]. Additionally, unrelieved pain stimulates glucagon and cortisol release, resulting in insulin resistance and hyperglycemia making the patient more porn to infection and complications [9, 10].

Furthermore, pain can make it difficult for patients to cough effectively, to breathe deeply, or to cooperate in physical therapy. It can also cause pulmonary secretions to be retained, which can result in consequences such as pulmonary atelectasis, pneumonia, and respiratory failure. These are considered serious complications after CBAG that might increase the length of stay (LoS) at the hospital postoperatively [11–13]. However, to our knowledge, no studies show a direct relationship between pain and LoS in patients with CABG.

Length of stay is considered by administrators and healthcare providers as a key indicator of postoperative recovery. Moreover, it is a crucial sign of long-term healing and a proxy measure of acute physical recovery, respectively [13–15]. Reduction of LoS post-CABG will not only result in improving the quality of life of the patients but also in reduction of the burden placed on the healthcare system due to postoperative care and managing these complications. Consequently, managing pain effectively for those patients is considered a key element in controlling these complications and in the reduction of LoS.

Pain management strategies fall under two major umbrellas: pharmacological (medicinal) and nonpharmacological (nonmedicinal) [2]. Medications like opioids are currently utilized to manage pain in cardiac patients, but their prices and side effects on many body systems and organs, which raise patient mortality and morbidity, make them undesirable as the first line of treatment [2, 16]. Recent studies about the use of opioids to treat pain and complications in other cardiac populations as acute myocardial infarction revealed that the use of such medication did not result in the reduction of the complication or in pain level. Based on that, it was recommended that pain should be treated by other modalities that address the underlying cause of the pain [17–19].

Researchers have investigated the effects of several nonpharmaceutical strategies for reducing pain post-CABG, such as music therapy, rapid relaxation [20], foot reflexology massage [21], cold therapy [10, 20, 22–24], deep breathing and relaxation exercise [9, 22, 24], and hearing a beloved person's voice [25]. However, to our knowledge, no research has been conducted specifically to check the effect of Holy Quran recitation on pain and LoS post-CABG.

Despite that, different studies concluded that Quran recitation was an effective treatment for diverse issues regarding health. For instance, specifically for patients with cardiac conditions, listening to the Holy Quran was found to improve sleep quality after cardiac surgeries [26], decrease levels of depression among patients undergoing CABG [27], and decrease levels of anxiety before cardiac catheterization procedure [28] and after acute myocardial infarction [29]. Among the noncardiac population, listening to the Holy Quran recital improved depressive and anxiety symptoms [30, 31] and decreased the number of smoked cigarettes and its withdrawal symptoms [32]. Among abdominal surgery patients, Quran recital decreased blood pressure, pulse rate, and respiratory rate as well as reduced patients' anxiety [33]. Additionally, listening to Holy Quran recitation has been found to stimulate alpha brain waves that are associated with the release of endorphins, enhancement of the stress threshold, creating a sense of relaxation, and mitigating of negative emotions that would decrease the postoperative pain levels [31]. For these reasons, this randomized control trial (RCT) was specifically designed to investigate the effect of listening to the Holy Quran recital on pain and LoS Post-CABG.

## 2. Methodology

### 2.1. Design, Sample, and Setting

To assess the impact of the interventions on the two groups (the intervention and the control), an RCT was carried out. The study population was composed of all eligible patients who were admitted to the study settings at the time of the study. Participants for this study were recruited using a consecutive sampling strategy. Every patient who visited any of the four main hospitals in Amman, Jordan, throughout the data collection period was sampled using this method by being checked for inclusion criteria. Up until the required sample size was reached, participants who met the inclusion criteria and consent to participate were included in the study. All patients who met the following inclusion criteria were included in the study: being at least 18 years old, having elective CABG surgery, being able to communicate with the researcher both during the intervention and the interview, being able to read and write Arabic, signed an informed consent form, and did not have any problem in hearing. Patients with hemodynamic instability were excluded from this study.

**Figure 1:**
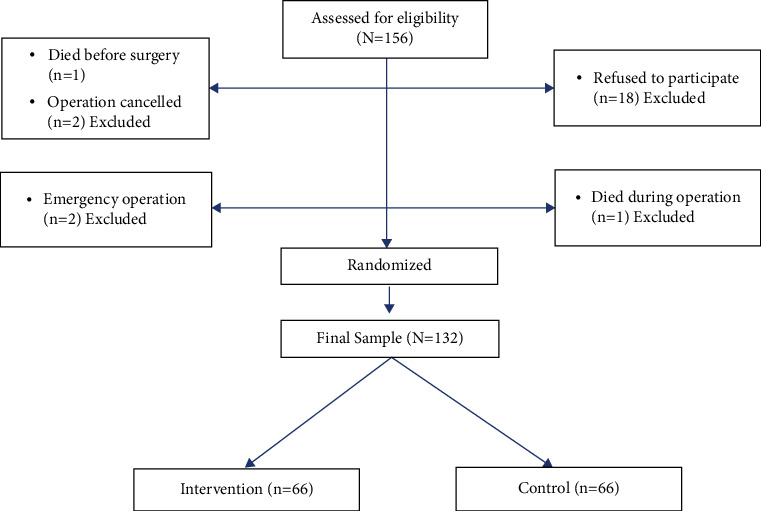
Flow diagram of participants.

G power software was used using the following presumptions, to ensure that sample size is adequate to obtain the statistical and clinical significance: (1) 0.05 alpha, (2) 0.8 power, (3) 0.5 medium effect size, (4) two-tailed test, and (5) the paired *t*-test and independent *t*-test as the statistical tests for analysis (see data analysis for more information). According to these presumptions, a paired *t*-test requires 34 participants per group, while an independent *t*-test requires 64 participants per group. A statistician used tables of random numbers to randomly allocate patients to intervention and control groups. The patients were given a sequential number. The researcher inserted it into a sealed, opaque envelope. The other researcher opened the package and then applied the positioning when it was time to position the patients. Due to the nature of the intervention, the nurses were not blinded to the allocation. The evaluation of the patient's results, however, was blinded. The RCT was carried out at four main hospitals in Amman, Jordan: two private, one educational, and one governmental (Figure 1).

### 2.2. Intervention

After being extubated and alert, the intervention group listened to the Holy Quran recited for 10 minutes twice daily at a time of 10 am and 2 pm for two days (often the second- and third-day postoperative). We chose Surah Al-Rehman because it is thought to be the most rhythmic surah in the Quran, and Qari Abdul Basit's recitation of it is both calming and powerful because it comes from the depths of his heart [34]. Each hospital's listing was based on disposable headphones for an iPad. Nurses were informed not to subject the intervention group of patients to other nonpharmacological pain relief measures to avoid the contamination of our results. The nurses provided routine treatment to the control group.

### 2.3. Measurement of Variables

#### 2.3.1. Sociodemographic and Clinical Characteristics

Age, gender, marital status, smoking status, and employment status of patients were assessed by patient interviews or by reviewing medical records (history of hypertension, history of diabetes mellitus, history of acute myocardial infarction, left ventricular ejection fraction, and body mass index).

#### 2.3.2. Pain and LoS

Pain was assessed at baseline and another time on day 4 postoperatively using a 0 to 10 pain numeric scale. LoS (in ICU and in the hospital) was abstracted from medical records after discharge and was reported in days.

#### 2.3.3. Ethical Considerations

Prior to collecting data, the project received approval from the IRB committee at the Applied Science Private University in Amman, Jordan, and from the chosen hospitals. The project was registered as a clinical trial (NCT05419554). Participants were given a thorough explanation of the study with assurances of anonymity, informed consent, and the freedom to leave at any moment without penalty. The informed consent form, which participants who accepted to participate in the study signed, allowed for the perusal of their medical records information. All data are maintained on a computer that has suitable coding and is password protected. This article makes use of aggregate data.

#### 2.3.4. Data Collection Process

At the selected sites, the coinvestigators met with cardiothoracic surgeons and nurse managers of the cardiothoracic surgery clinics to discuss the study. The coinvestigators contacted all patients scheduled for elective CABGs and evaluated their eligibility. Patients were informed about the trial and, if they consented to take part, signed an informed consent form before being randomly assigned to either an intervention group or a control group.

The participants were interviewed by the coinvestigators and given the opportunity to complete the sociodemographic survey and pain scale on the second postoperative day. Additionally, the intervention group received the intervention on that day as previously indicated. The intervention was repeated for the intervention group on the third day, while the control group continued to receive standard care. The patients in both groups recompleted the pain scale on the fourth postoperative day in order to compare results. After discharge, LoS was collected from medical records and was reported in days.

### 2.4. Data Analysis

Data were analyzed using SPSS version 25. Descriptive statistics were used to describe the sociodemographic and clinical characteristics of the sample. To check if the intervention has an effect on pain levels, the following steps were done: first, to rule out differences and prevent bias, the baseline pain levels between the two groups before the intervention began were compared using an independent samples *t*-test, second: after the intervention, the levels of pain were compared between the two groups using an independent samples *t*-test, and third: a paired *t*-test was used to compare the pain levels in the intervention group between pre- and postintervention. To check if the intervention has an effect on the LoS, the LoS between the two groups was compared using an independent *t*-test.

## 3. Results

A total of 132 patients participated in this RCT, 66 per each group. The mean age was around 67 years, and more than half of the samples were males. Around three-quarters of the sample had hypertension, which was the only difference between the intervention and the control group with regard to sociodemographic and clinical characteristics. The intervention group had more smokers than the control group. Other characteristics are presented in Table 1.

There was no statistical difference in the pain levels at the baseline between the two groups. However, the intervention group had lower levels of pain compared to the control group at the follow-up (Table 2).

Paired *t*-test testing the pain level for the intervention group prior to the intervention and after the intervention showed that there was a significant reduction in the pain level, (M [SD], 6.82 [2.27] vs. 4.65 [2.18], *t* = 23.65, *p* < 0.001). In addition, the intervention group had shorter LoS in the ICU and in the hospital compared to the control group (Table 3).

## 4. Discussion

Current pain theories explain pain as a physical, psychological, and social experience [35]. From this perspective, pain management should not rely only on painkillers; instead, nonpharmacological measures that focus on psychological and spiritual aspects should be a part [9]. Music therapy is one of the nonpharmacological measures recently used for pain relief among post-CABG patients [36]. In Jordan, listening to the Holy Quran is preferable, especially during the illness period due to the spiritual, religious, and cultural background.

Considering the high prevalence of pain among patients' post-CABG [37], its deleterious outcomes [38], and the effective role of spiritual care in reliving the pain [39], the current study was implemented to investigate the effect of listening to Holy Quran recital on pain and LoS among post-CABG Jordanian patients.

The findings of the current study supported the hypothesis geared towards investigating the positive effect of listening to the Holy Quran recital on pain and LoS post-CABG. Since there is a statistically significant difference between the intervention and control group of patients, improvements in the intervention group can be attributed to the effect of listening to the Holy Quran post-CABG, taking into consideration that randomization of patients made the effect of other confounding (systemic bias) minimal.

Recitation of the Holy Quran could soothe patients' hearts, heal their pains, and enhance mental health. Listening to the Holy Quran reduces the pain signal transmission to the central nervous system, leading to the relaxation of muscles, and diverting the mind from the pain. Moreover, it stimulates alpha brain waves which increases endorphin levels, enhances the stress threshold, creates a sense of relaxation, and then relieves pain [31]. Pain and LoS could be interrelated. Because of sternal pain, the patient's breathing becomes superficial. Atelectasis and respiratory insufficiency then exist [40], and these complications could lengthen the ICU stay. This may explain the significant effect of listening to the Holy Quran recital on pain and LoS post-CABG in the intervention group.

The pain level improvement started in the follow-up assessment for patients in the intervention group. Researchers [39, 41, 42] highlighted in their studies the pure effect of listening to the Holy Quran on pain relief, as explained by the statistically significant differences between the two groups of patients. Different nonpharmacological measures were investigated in previous studies, for example, cold therapy [43], deep breathing exercises [2], massage therapy [44], and acupressure [45]. These studies showed an improvement in the pain severity between the pre- and postintervention. In comparison to these studies, the use of the Holy Quran as a nonpharmacological intervention carries no risks, takes no time, and accounts for zero load on ICU nurses.

In terms of LoS, the current study findings indicated that patients in the intervention group had shorter LoS in the ICU and the hospital compared to those in the control group. In other words, the findings of the current study reflected a significant difference in the number of days the patients stayed in both the ICU and hospital between both groups of patients in favor of the intervention group. The longer the ICU/hospital stays, the higher the risk for patient's postoperative complications such as healthcare-acquired infections and increased readmission rate [46]. Longer stays in ICU/hospital post-CABG could lead to lower bed capacities and increased costs. According to the Agency for Health Care Research and Quality, the average hospital stay is 4.5 days worldwide [47]. However, previous Jordanian studies show that the LoS is much higher [13, 48, 49]. In the present study findings, the mean length of ICU and hospital stay was high compared to some studies [50, 51] and low compared to other studies [43, 52].

Since patients' outcomes, i.e., less pain intensity and shorten LoS in the intervention group, were significantly better than those in the control group, nurse managers and clinicians would encourage spiritual care including listening to recitation to the Holy Quran for patients following post-CABG. Further studies are needed to compare the effect of listening to recitation to the Holy Quran on the pain level associated with different painful procedures, for example, removal of chest tube, use of incentive spirometry, and breathing and coughing exercises. Because pain is a subjective experience and feeling that could be affected by multiple variables, future studies better include the patients' subjective experience qualitatively. Also, to avoid the effect of social, psychological, or personal factors on the effect of the intervention, the use of the crossover method in future studies could increase the power of the study.

### 4.1. Limitations of the Study

Despite the homogeneity of the two groups, there are several factors that could affect the pain perception between patients in both groups. Pain experiences were not qualitatively examined, which might guide the ICU clinician in improving the delivered care.

## 5. Conclusion

The present study revealed that listening to the Quran was significantly effective in improving pain intensity among post-CABG patients and shortening their hospital/ICU stay. Based on these findings, listening to the Quran recitations could be used to decrease the need for analgesics and sedatives among post-CABG patients. Further research is recommended to examine the interval, timing, and duration that listening to the Quran needs to be applied to optimize clinical benefits. Moreover, studies might compare the effect of listening to the Quran combined with other spiritual interventions post-CABG and also evaluate the pain experience using quantitative methods, subjectively and objectively, and qualitative ones to provide the clinicians with the full picture regarding the use of the intervention.

## Figures and Tables

**Table 1:** Patient characteristics, intervention, and control groups. (*N* = 132).

Characteristics	*N* (%) or mean ± SD			*t* or Chi-square	*P*
	Total sample	Intervention (*n* = 66)	Control (*n* = 66)		
Age	67.1 ± 9.6	67.7 ± 8.3	66.5 ± 10.7	0.6	NS
Gender					
Male	69 (52.3)	38 (57.6)	31 (47.0)	1.1	NS
Female	63 (47.7)	28 (42.4)	35 (53.0)		
Marital status					
Married	83 (62.9)	44(66.7)	39 (59.1)	2.7	NS
Single/divorced/widowed	49 (37.1)	22 (33.3)	27 (40.9)		
History of smoking					
Never smoked	41 (31.1)	21 (31.8)	20 (20.3)	2.3	NS
Current smoker	37 (28.0)	20 (30.3)	17 (25.8)		
Former smoker	54 (40.9)	25 (37.9)	29 (43.9)		
History of HTN	100 (75.8)	55 (83.3)	45 (68.1)	3.9	0.04
History of DM	60 (45.6)	34 (51.5)	26 (39.4)	0.1	NS
History of HF	34 (65.0)	17 (25.8)	17 (25.8)	0.002	NS
LVEF	46.2 ± 8.0	46.3 ± 8.5	46.1 ± 7.6	0.4	NS
BMI (kg/m^2^)	26.1 ± 4.9	26.2 ± 5.5	25.9 ± 4.2	0.5	NS

BMI: body mass index, DM: diabetes miletus, HTN: hypertension, LVEF: left ventricular ejection fraction, NS: not significant, SD: standard deviation.

**Table 2:** Independent sample *t*-tests between intervention and control groups at baseline and follow-up measurements.

Outcome	Group	M ± SD	*t*	Sig (2-tailed)
Baseline measurement				
Pain level	Intervention	6.82 ± 2.27	−1.42	NS
	Control	7.31 ± 2.11		

Follow-up measurement				
Pain level	Intervention	4.65 ± 2.18	−2.44	<0.05
	Control	5.45 ± 2.01		

NS: not significant.

**Table 3:** Independent sample *t*-tests between intervention and control groups regarding LoS in ICU and hospital.

Outcome	Intervention M ± SD	Control M ± SD	*t*	Sig (2-tailed)
ICU LoS	5.0 ± 4.02	6.38 ± 4.18	−2.01	<0.05
Hospital LoS	10.15 ± 9.21	15.01 ± 13.14	−2.60	<0.05

ICU: intensive care unit, LoS: length of stay.

## Data Availability

Derived data supporting the findings of this study are available from the corresponding author (Fatma Refaat Ahmed) upon request.

## Additional Points

This trial is registered with Clinicaltrials.gov NCT05419544.

## References

[1] Panagopoulou E., Montgomery A., Benos A. (2006). Quality of life after coronary artery bypass grafting: evaluating the influence of preoperative physical and psychosocial functioning. *Journal of Psychosomatic Research*.

[2] Babamohamadi H., Karkeabadi M., Ebrahimian A. (2021). The effect of rhythmic breathing on the severity of sternotomy pain after coronary artery bypass graft surgery: a randomized controlled clinical trial. *Evidence-based Complementary and Alternative Medicine*.

[3] AbuRuz M. E., Al-Dweik G. (2022). Depressive symptoms, perceived control and quality of life among patients undergoing coronary artery bypass graft: a prospective cohort study. *BMC Nursing*.

[4] Montrief T., Koyfman A., Long B. (2018). Coronary artery bypass graft surgery complications: a review for emergency clinicians. *American Journal of Emergency Medicine*.

[5] Hussain S. M. A., Harky A. (2019). Complications of coronary artery bypass grafting. *International journal of medical reviews*.

[6] Zubrzycki M., Liebold A., Skrabal C. (2018). Assessment and pathophysiology of pain in cardiac surgery. *Journal of Pain Research*.

[7] Jahangirifard A., Razavi M., Ahmadi Z. H., Forozeshfard M. (2018). Effect of TENS on postoperative pain and pulmonary function in patients undergoing coronary artery bypass surgery. *Pain Management Nursing*.

[8] Dunwoody C. J., Krenzischek D. A., Pasero C., Rathmell J. P., Polomano R. C. (2008). Assessment, physiological monitoring, and consequences of inadequately treated acute pain. *Pain Management Nursing*.

[9] Jarrah M. I., Hweidi I. M., Al-Dolat S. A. (2022). The effect of slow deep breathing relaxation exercise on pain levels during and post chest tube removal after coronary artery bypass graft surgery. *International journal of nursing sciences*.

[10] Mohammadi N., Pooria A., Yarahmadi S. (2018). Effects of cold application on chest tube removal pain in heart surgery patients. *Tanaffos*.

[11] Fiorelli A., Morgillo F., Milione R. (2012). Control of post-thoracotomy pain by transcutaneous electrical nerve stimulation: effect on serum cytokine levels, visual analogue scale, pulmonary function and medication. *European Journal of Cardio-Thoracic Surgery*.

[12] Fibla J. J., Molins L., Mier J. M., Hernandez J., Sierra A. (2015). A randomized prospective study of analgesic quality after thoracotomy: paravertebral block with bolus versus continuous infusion with an elastomeric pump. *European Journal of Cardio-Thoracic Surgery*.

[13] AbuRuz M. E. (2019). Pre-operative depression predicted longer hospital length of stay among patients undergoing coronary artery bypass graft surgery. *Risk Management and Healthcare Policy*.

[14] Poole L., Kidd T., Leigh E., Ronaldson A., Jahangiri M., Steptoe A. (2014). Depression, C-reactive protein and length of post-operative hospital stay in coronary artery bypass graft surgery patients. *Brain, Behavior, and Immunity*.

[15] Poole L., Leigh E., Kidd T., Ronaldson A., Jahangiri M., Steptoe A. (2014). The combined association of depression and socioeconomic status with length of post-operative hospital stay following coronary artery bypass graft surgery: data from a prospective cohort study. *Journal of Psychosomatic Research*.

[16] Erstad B. L., Puntillo K., Gilbert H. C. (2009). Pain management principles in the critically ill. *Chest*.

[17] Parodi G., Bellandi B., Xanthopoulou I. (2015). Morphine is associated with a delayed activity of oral antiplatelet agents in patients with ST-elevation acute myocardial infarction undergoing primary percutaneous coronary intervention. *Circulation. Cardiovascular interventions*.

[18] AbuRuz M. E. (2016). The effect of pain and morphine use on complication rates after acute myocardial infarction. *Health Science Journal*.

[19] Abu Taha A., AbuRuz M. E., Momani A. (2022). Morphine use did not eliminate the effect of pain on complications after acute myocardial infarction. *The Open Nursing Journal*.

[20] Demir Y., Khorshıd L. (2010). The effect of cold application in combination with standard analgesic administration on pain and anxiety during chest tube removal: a single-blinded, randomized, double-controlled study. *Pain Management Nursing*.

[21] Chandrababu R., Rathinasamy E. L., Suresh C., Ramesh J. (2019). Effectiveness of reflexology on anxiety of patients undergoing cardiovascular interventional procedures: a systematic review and meta-analysis of randomized controlled trials. *Journal of Advanced Nursing*.

[22] Ayyasi M., Ghafari R., Yazdani J., Gorji H., Nesami B. (2014). Comparison of ice packs application and relaxation therapy in pain reduction during chest tube removal following cardiac surgery. *North American Journal of Medical Sciences*.

[23] Dave K. (2016). Effects of cold application on pain and anxiety during chest tube removal among post operative cardiac surgery adult patients. *International Journal of Advances in Nursing Management*.

[24] Mohamed El Mokadem N., Shimaa E., Ibraheem S. (2017). Cold application and breathing exercises to reduce pain and anxiety during chest tube removal. *American Journal of Nursing Science*.

[25] Salmani F., Abadi A., Taheri S. M., Majd H. A., Abbaszadeh A. (2017). Effect of beloved person’s voice on chest tube removal pain in patients undergoing open heart surgery: fuzzy logistic regression model. *Archives of Advances in Biosciences*.

[26] Mousavi F., Gholizadeh B., Rahimi A., Heidari M. R. (2019). The effect of the holy quran voice on improving sleep quality of patients after cardiac surgery. *Journal of Critical Care Nursing*.

[27] Tajbakhsh F., Hosseini M., Fallahi-Khoshknab M., Rokofian A., Rahgozar M., Mary Davidson P. (2018). The effect of spiritual care on depression in patients following coronary artery bypass surgery: a randomized controlled trial. *Religions*.

[28] Babaii A., Abbasinia M., Hejazi S. F., Seyyed Tabaei S. R., Dehghani F. (2015). The effect of listening to the voice of Quran on anxiety before cardiac catheterization: a randomized controlled trial. *Health, Spirituality and Medical Ethics*.

[29] Najafi Z., Tagharrobi Z., Taghadosi M., Sharifi K., Farrokhian A. (2014). The effect of simultaneous aromatherapy and quran recitation on anxiety level of patients with myocardial infarction. *Complementary Medicine Journal of Faculty of Nursing and Midwifery*.

[30] Mahjoob M., Nejati J., Hosseini A., Bakhshani N. M. (2016). The effect of holy quran voice on mental Health. *Journal of Religion and Health*.

[31] Ghiasi A., Keramat A. (2018). The effect of listening to holy quran recitation on anxiety: a systematic review. *Iranian Journal of Nursing and Midwifery Research*.

[32] Maziha Z., Imran A., Azlina I., Harmy M. Y. (2018). Randomized controlled trial on the effect of Al-Quran recitation vs counseling on smoking intensity among Muslim men who are trying to quit smoking. *Malaysian Family Physician*.

[33] Mirbagher Ajorpaz N., Aghajani M., Shahshahani M. S. (2011). The effects of music and Holy Quran on patient’s anxiety and vital signs before abdominal surgery. *Evidence Based Care*.

[34] Rafique R., Anjum A., Raheem S. S. (2019). Efficacy of surah Al-rehman in managing depression in muslim women. *Journal of Religion and Health*.

[35] Moayedi M., Davis K. D. (2013). Theories of pain: from specificity to gate control. *Journal of Neurophysiology*.

[36] Dai W. S., Huang S. T., Xu N., Chen Q., Cao H. (2020). The effect of music therapy on pain, anxiety and depression in patients after coronary artery bypass grafting. *Journal of Cardiothoracic Surgery*.

[37] Harrogate S. R., Cooper J. A., Zawadka M., Anwar S. (2021). Seven-year follow-up of persistent postsurgical pain in cardiac surgery patients: a prospective observational study of prevalence and risk factors. *European Journal of Pain*.

[38] Krakowski J. C., Hallman M. J., Smeltz A. M. (2021). Persistent pain after cardiac surgery: prevention and management. *Seminars in Cardiothoracic and Vascular Anesthesia*.

[39] Imran M., Gul R. B., Batool S. (2021). Effects of Surah Al-Rehman on pain, oxygen-saturation, and vital signs in post CABG patients: a Pilot Study. *Journal of Shifa Tameer-e-Millat University*.

[40] Zarrizi M., Paryad E., Khanghah A. G., Leili E. K., Faghani H. (2021). Predictors of length of stay in intensive care unit after coronary artery bypass grafting: development a risk scoring system. *Brazilian Journal of Cardiovascular Surgery*.

[41] Nasiri M., Fayazi S., Ghaderi M., Naseri M., Adarvishi S. (2014). The effect of reciting the word “allah” on pain severity after coronary artery bypass graft surgery: a randomized clinical trial study in Iran. *Anesthesiology and Pain Medicine*.

[42] Aisyah P. S., Sofiyah Y., Pangestuty E., Pangestuty E. (2019). The effect of the sound of Holy Quran on pain level of neonates during invasive procedure. *KnE Life Sciences*.

[43] Seweid M. M., Ahmed N. T., Ramadan B. A., Ahmed F. R. (2021). Effect of cold application on incisional pain associated with incentive spirometry after coronary artery bypass graft surgery. *International Journal of Africa Nursing Sciences*.

[44] Boitor M., Martorella G., Maheu C., Laizner A. M., Gélinas C. (2018). Effects of massage in reducing the pain and anxiety of the cardiac surgery critically ill-a randomized controlled trial. *Pain Medicine*.

[45] Sen S., Aygin D. (2019). A randomized trial of acupressure on pain management after cardiac surgery. *International Journal of Clinical and Experimental Medicine*.

[46] Oshvandi K., Pakrad F., Mohamadi Saleh R., Seif Rabiei M. A., Shams A. (2020). Post-operative symptoms and complications in patients having undergone coronary artery bypass graft in hamadan: a descriptive cross-sectional study. *Jundishapur Journal of Chronic Disease Care*.

[47] Awad A., Bader-El-Den M., McNicholas J. (2017). Patient length of stay and mortality prediction: a survey. *Health Services Management Research*.

[48] Abu-Humaidan A. H., Ahmad F. M., Al-Binni M. A., Bani Hani A., Abu Abeeleh M. (2021). Characteristics of adult sepsis patients in the intensive care units in a tertiary hospital in Jordan: an observational study. *Critical care research and practice*.

[49] Bani Hani D. A., Alshraideh J. A., Alshraideh B. (2022). Patients’ experiences in the intensive care unit in Jordan: a cross-sectional study. *Nursing Forum*.

[50] Kao K. D., Lee S. Y. K. C., Liu C. Y., Chou N. K. (2022). Risk factors associated with longer stays in cardiovascular surgical intensive care unit after CABG. *Journal of the Formosan Medical Association*.

[51] Techane T., Nigussa E., Lemessa F., Fekadu T. (2022). Factors associated with length of intensive care unit stay following cardiac surgery in cardiac center Ethiopia, addis ababa, Ethiopia: institution based cross sectional study. *Research Reports in Clinical Cardiology*.

[52] Widyastuti Y., Stenseth R., Wahba A., Pleym H., Videm V. (2012). Length of intensive care unit stay following cardiac surgery: is it impossible to find a universal prediction model?. *Interactive Cardiovascular and Thoracic Surgery*.

